# Nightmares share genetic risk factors with sleep and psychiatric traits

**DOI:** 10.1038/s41398-023-02637-6

**Published:** 2024-02-27

**Authors:** Hanna M. Ollila, Nasa Sinnott-Armstrong, Katri Kantojärvi, Martin Broberg, Teemu Palviainen, Samuel Jones, Vili Ripatti, Anita Pandit, Robin Rong, Kati Kristiansson, Nils Sandman, Katja Valli, Christer Hublin, Samuli Ripatti, Elisabeth Widen, Jaakko Kaprio, Richa Saxena, Tiina Paunio

**Affiliations:** 1 Institute for Molecular Medicine Finland (FIMM), University of Helsinki, Helsinki, Finland; 2 Center for Genomic Medicine, Massachusetts General Hospital, Boston, MA USA; 3 Program in Medical and Population Genetics, Broad Institute, Cambridge, MA USA; 4 Department of Anesthesia, Critical Care and Pain Medicine, Massachusetts General Hospital and Harvard Medical School, Boston, MA USA; 5 Department of Genetics, School of Medicine, Stanford University, Stanford, CA USA; 6 Population Health, Finnish Institute for Health and Welfare, Helsinki, Finland; 7 Department of Psychiatry and SleepWell Research Program, Faculty of Medicine, University of Helsinki and Helsinki University Central Hospital, Helsinki, Finland; 8 Department of Biostatistics, School of Public Health, University of Michigan, Ann Arbor, MI 48109 USA; 9 Department of Psychology and Speech-Language Pathology, and Turku Brain and Mind Center, University of Turku, Turku, Finland; 10 Department of Cognitive Neuroscience and Philosophy, University of Skövde, Skövde, Sweden; 11 Finnish Institute of Occupational Health, Helsinki, Finland; 12 Division of Sleep and Circadian Disorders, Brigham and Women’s Hospital and Harvard Medical School, Boston, MA USA

**Keywords:** Genomics, Psychiatric disorders

## Abstract

Nightmares are vivid, extended, and emotionally negative or negative dreams that awaken the dreamer. While sporadic nightmares and bad dreams are common and generally harmless, frequent nightmares often reflect underlying pathologies of emotional regulation. Indeed, insomnia, depression, anxiety, or alcohol use have been associated with nightmares in epidemiological and clinical studies. However, the connection between nightmares and their comorbidities are poorly understood. Our goal was to examine the genetic risk factors for nightmares and estimate correlation or causality between nightmares and comorbidities. We performed a genome-wide association study (GWAS) in 45,255 individuals using a questionnaire-based assessment on the frequency of nightmares during the past month and genome-wide genotyping data. While the GWAS did not reveal individual risk variants, heritability was estimated at 5%. In addition, the genetic correlation analysis showed a robust correlation (rg > 0.4) of nightmares with anxiety (rg = 0.671, *p* = 7.507e−06), depressive (rg = 0.562, *p* = 1.282e−07) and posttraumatic stress disorders (rg = 0.4083, *p* = 0.0152), and personality trait neuroticism (rg = 0.667, *p* = 4.516e−07). Furthermore, Mendelian randomization suggested causality from insomnia to nightmares (beta = 0.027, *p* = 0.0002). Our findings suggest that nightmares share genetic background with psychiatric traits and that insomnia may increase an individual’s liability to experience frequent nightmares. Given the significant correlations with psychiatric and psychological traits, it is essential to grow awareness of how nightmares affect health and disease and systematically collect information about nightmares, especially from clinical samples and larger cohorts.

## Introduction

Nightmares are vivid, extended, and extremely dysphoric dreams that usually include themes involving threats to survival, security, physical integrity, or self-esteem and cause the dreamer to wake up (American Academy of Sleep Medicine). While sporadic nightmares and bad dreams are common and generally harmless, frequent or very intensive nightmares may reflect an underlying pathology in the processing of emotions. While almost everyone experiences sporadic nightmares, experiencing frequent nightmares is relatively rare, at less than 5% of the overall population, with higher prevalence in females compared to males and a higher prevalence of nightmares in childhood compared to adulthood [1–3].

While the risk factors behind nightmares are not well understood, nightmares can be caused by negative experiences and fear, such as traumatic events, as observed in war veterans with PTSD [3]. Our earlier epidemiological studies and those by others have shown that both sleep problems and severe sleep disorders, such as insomnia and narcolepsy, are associated with nightmares [4–7]. Furthermore, we have previously shown a familial aggregation between nightmares and psychiatric traits, and alcohol use in general [2]. Similarly, we and others have previously shown that nightmares correlate with sleep and psychiatric or behavioral traits, including alcohol use, insomnia symptoms, overall pain, including headaches, and a core feature of depressed mood, specifically a negative attitude toward the self [2, 3, 8, 9]. However, the biological basis behind nightmares and the causality between nightmares and psychiatric traits have not been thoroughly established.

**Fig. 1:**
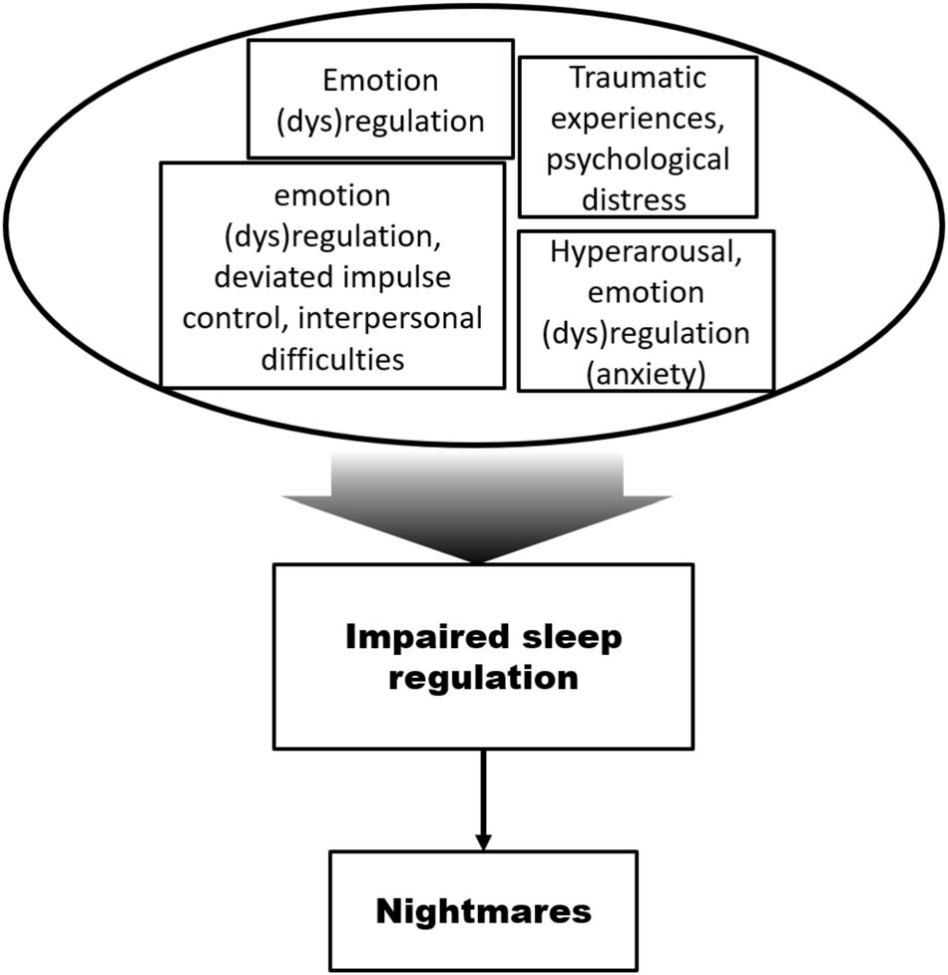
Multiple traits affecting mood and psyche contribute to nightmares. A model demonstrating how psychiatric trait affect nightmare incidence in part via impaired sleep regulation and hyperarousal interfering with REM sleep.

Previously, in a longitudinal study on war veterans with PTSD, it was demonstrated that both insomnia and nightmares had a strong association with PTSD, but symptom insomnia did not resolve spontaneously, whereas the prevalence and severity of nightmare symptoms diminished modestly over time [10, 11]. This finding suggests an independent component of insomnia in PTSD [10]. In the clinical setting, nightmares tend to remain unreported, and people underestimate the frequency of nightmares in retrospective analysis versus daily logs [12]. Consequently, the overall clinical relevance of nightmares has possibly been underestimated despite their known epidemiological association with a large number of sleep disorders, psychiatric disorders, and even suicide [13]. Overall, hyperarousal, as a result from emotional stress and problems with sleep regulation have been linked to many psychiatric traits including but not limited to personality disorders, psychosis, mood disorders, PTSD, and insomnia, which also demonstrate comorbidity and overlap with each other [14–17], we show an overview model of the effects on nightmares in Fig. 1.

Interindividual genetic differences account for 36 to 51% of the variance in liability to nightmares in the population [2]. Genetics offers an opportunity to identify new predisposing loci and tools to understand whether underlying biological mechanisms are shared with psychiatric traits. Indeed, while these earlier studies sketch a pathway in which nightmares overlap with psychiatric and sleep traits, it has remained inconclusive whether these other traits, such as mood disorders or insomnia, are a cause or consequence of nightmares. In addition to genetic correlation, Mendelian randomization (MR) offers a tool to explore causality between the frequency of nightmares and related psychiatric and sleep traits.

In this study, our goal was to examine the underlying biological and epidemiological mechanisms that affect the frequency of nightmares. Our aim was to understand the putative causal links between nightmares as well as sleep and psychiatric traits. Given that nightmares are heritable, we used a genome-wide association study design (GWAS) in 45,255 individuals with a self-reported frequency of nightmares to elucidate the underlying biology as well as shared mechanisms and links with psychiatric diseases. As our cohorts also included individual alcohol consumption information, we decided to perform a sensitivity analysis where we removed individuals with significant alcohol consumption as it may influence nightmare incidence [6, 13].

## Materials and methods

### Study populations

A total of 45,255 individuals with full information on nightmare frequency and genotyping participated in this study. Studies outlined in detail below included Finrisk 1997, 2002, 2007, and 2012 (*N* = 21,243), the Finnish Twin Cohort Study (*N* = 5556), FinHealth (*N* = 10,049), GeneRISK (*N* = 6610) and Genes for Good (*N* = 1367). The individuals contributed genetic and questionnaire data and provided written informed consent for research participation.

#### FINRISK (*n* = 21,243)

This cohort comprises health surveys, collected every five years, from random cross-sectional population samples of Finnish adults. The data are collected in the form of health questionnaires and a formal health examination at a local health care center. In the Finrisk dataset, nightmares were assessed with the question “During the past 30 days have you had nightmares?”. The response options were “often”, “sometimes”, and “never.” Other measures, including age, gender, and the survey year in which the subject took part (Finrisk surveys 1992, 1997, 2002, or 2007) as well as alcohol use were assessed. Alcohol intake was measured using a question on the frequency of alcohol intoxication. Nightmares were used as a linear trait from 1 to 3, with 1 being no nightmares and 3 frequent nightmares. The genotyping was done with the HumanOmniExpress array at Wellcome Trust Sanger Institute (Cambridge, UK), at the Broad Institute of Harvard and MIT (MA, USA), and at the Institute for Molecular Medicine Finland (FIMM) Genotyping Unit. Imputation was performed with SHAPEIT2 and IMPUTEv2 using a custom haplotype set of 2000 whole genome sequenced Finnish individuals and 1000 Genomes project phase 3 haplotypes as reference panels. Genome-wide association analysis was conducted using SNPtest with the option *score* in the SNPtest package [18]. Analyses were adjusted for relevant covariates comprising age, gender, and identity by descent-calculated genetic correlation matrix with the top 10 principal components, the survey year, genotyping chip, and the cohort for each Finrisk sub cohort. In addition, individuals who were intoxicated once or more often per month were removed from the secondary analysis, because frequent heavy use of alcohol has been previously found to increase the risk of frequent nightmares [6].

#### GeneRISK (*n* = 6610)

This is a prospective study consisting of 7342 individuals. A total of 6610 individuals with full genotype information who had answered the nightmare question were available after the genotyping QC. Individuals aged 45–65 years old were recruited between 2015–2017 from southern Finland. These participants underwent a health check-up, and information on their lifestyles and earlier health was recorded via electronic questionnaires. Nightmares were assessed with the question “During the past 30 days have you had nightmares?”. The response options were “often”, “sometimes”, and “never.” Alcohol intake was measured using a question on the frequency of alcohol intoxication. Genotyping was performed using HumanCoreExome BeadChip (Illumina Inc., San Diego, CA, USA). Genotypes were called using GenomeStudio and zCall at the Institute for Molecular Medicine Finland (FIMM), phased using SHAPEIT2, and imputed using IMPUTE2 and a combined reference panel of 1000 Genomes Phase I integrated haplotypes and 1943 Finnish genomes [19]. An association analysis was computed using Plink and adjusted for age, sex, and principal components to account for the population structure.

#### National FinHealth 2017 study (*n* = 10,049)

The national FinHealth 2017 study comprises a random sample of adults in Finland, and it focuses on objective and perceived health, quality of life, lifestyle, prevalence, and risk factors for common health problems including sleep, insomnia, and alcohol consumption. Nightmares were assessed with the question “During the past 30 days have you had nightmares?”. The response options were “often”, “sometimes”, and “never.” Alcohol intake was measured using a question on the frequency of alcohol intoxication. The study combines questionnaires from earlier Health 2000–2011 and Finrisk collections. Similar to GeneRISK, genotyping was performed using HumanCoreExome BeadChip (Illumina Inc., San Diego, CA, USA). Genotypes were called using GenomeStudio and zCall at the Institute for Molecular Medicine Finland (FIMM), phased using SHAPEIT2, and imputed using IMPUTE2 and a combined reference panel of 1000 Genomes Phase I integrated haplotypes and 1943 Finnish genomes.

#### Finnish twin cohort (*n* = 5556)

The study cohort consists of same-sexed twin pairs born before 1958 who participated in two questionnaire surveys in 1975 and 1981. In 1990, twins who had participated in either previous survey and who were born between 1930 and 1957 were invited to participate in a questionnaire survey [20]. The 1990 survey included a broad set of items on the frequency of parasomnias in childhood and adulthood, as reported earlier [2, 21]. In the Finnish Twin Cohort, two questions assessed the frequency of nightmares in childhood and adulthood [4]. In the analysis, we used the data on nightmares in the adults. For adults, the question was “How often have the following nighttime symptoms been present in adulthood?” The choices were weekly, about once a month, less often, never, and cannot say. Those with weekly nightmares were defined as cases, and those who had nightmares never, rarely, or monthly were defined as controls. Heavy drinking occasions were defined as consuming at least once a month and on a single occasion, more than five beers, a bottle of wine or a half-bottle of spirits (or a similar amount), to correspond to five standard drinks or >60 g of pure alcohol on a single occasion [22]. For the Finnish Twin Cohort, we used the Haplotype Reference Consortium release 1.1 reference panel [23] for genotype imputation using a protocol provided by the University of Michigan Imputation Server [24]. For the Finnish Twin Cohort, primary analyses were conducted using RVTESTS [25] with a linear mixed model regression using a score test for testing association. The sample relatedness and population stratification were taken into account in the genetic relatedness matrix used as a random effect of the model.

#### Genes for Good (*n* = 1797)

Genes for Good is an online genetics study conducted through a Facebook web app. Participants self-report health and behavioral data through online surveys and submit a saliva sample in the mail for genotyping. The sample includes participants over 18 years of age from all 50 U.S. states. Altogether, 1797 individuals in Genes for Good participated in the study. In Genes for Good, the frequency of nightmares was assessed with the question: “During the last 30 days, how often have you had nightmares? Nightmares are dreams that evoke such strong negative feelings that they wake you up.” The answer options were “1. Almost every morning, 2. Several times a week, 3. Once a week, 4. Two or three times a month, 5. Once a month, 6. I did not have nightmares that woke me up during the last month”. The phenotypes were harmonized in a manner similar to those in the Finnish Twin cohort: those with nightmares at least once a week were defined as cases, and those with nightmares never, rarely, or less than weekly were defined as controls. The analysis was computed as logistic mixed model regression with SAIGE [26], adjusting for age, sex, and principal components. In the secondary analysis, because information on how much alcohol individuals consumed at one occasion was not available, the use of alcohol was adjusted instead as the number of drinks people had had during the last 30 days. After quality control and filtering, we used a total of 1367 individuals for the meta-analysis.

#### Meta-analysis

*P* value-based meta-analysis using METAL [27, 28] was used for the analysis across all cohorts (N individuals in all cohorts = 45,255). To produce comparable effect estimates and standard errors and the largest possible sample for downstream Mendelian randomization analysis, we computed a secondary meta-analysis with Finrisk, FinHealth 2017, and GeneRisk cohorts (*N* = 37,902 individuals).

#### Genetic correlation

To estimate the genetic correlation between traits, we used LD score regression, which considers linkage disequilibrium (LD) (e.g., correlation due to close proximity at the DNA chain) between genetic variants. Similarly, trait heritability explained by common variants and tissue-specific partitioned heritability were estimated using LD score regression [29]. The *P* values were Bonferroni-corrected.

#### Mendelian randomization

Mendelian randomization was computed between nightmares and those traits that have been observed in previous epidemiological studies and those that showed a significant genetic correlation with nightmares. We used the summary statistics from the secondary meta-analysis (using only Finrisk, FinHealth, and GeneRisk cohorts) for nightmares as the outcome in MR, because the GWAS analysis by Genes for Good and the Finnish Twin Cohort were not performed using a three-level variable for nightmares. An analysis was performed using the MRCIEU/TwoSampleMR package in R 3.5.0 [30]. We calculated inverse variance weighted and MR-Egger models to estimate the effect sizes. For the associations with significant effects, we estimated pleiotropy as implemented in the MRinstruments R package.

#### Polygenic risk score analysis

A polygenic risk score analysis was performed using PRScs [31] to estimate polygenic weights for the variants in the nightmare meta-analysis summary statistics, using the 1000 Genomes European Panel as the LD reference panel and the UK Biobank (UKB) cohort as the test panel (*N* = 502,459). After the weight estimation, we performed polygenic scoring using PLINK 2 [32]. The polygenic risk scores for nightmares were then Z-normalized and tested in a linear model regression analysis using R against the UKB end-point 2050 (frequency of depressed mood in the last 2 weeks), 4526 (happiness), using age, sex, and Principal Components (PC)1–10 as covariates. We tested the differences between traits using the likelihood ratio test as implemented in the R package *lmtest*.

## Results

### Meta-analysis of GWAS of nightmares

In the current study, we explored genetic risk factors that contribute to the frequency of nightmares and that possibly connect nightmares with psychiatric traits; we have extensively studied the epidemiological correlates of nightmares earlier in the cohorts examined here [3, 4, 6, 8]. The overall SNP-based heritability was 5% [SNPh^2^ = 0.05, se = 0.017]. Because previous studies show that heavy alcohol use correlates with nightmares [6, 33], we performed a GWAS after controlling for age and sex (nightmares adjusted for baseline covariates) and removed individuals who were intoxicated at least once per month (nightmares in individuals with no indication of heavy alcohol use and adjusting for covariates). While we observed an association in the GWAS, in the largest Finrisk cohort at the *MYOF* locus in the alcohol-adjusted model (rs701873, beta = 0.034, se = 0.0059, *p* = 1.5e−8), the global meta-analysis across all cohorts did not reveal consistent associations across all cohorts with either model (Supplementary Fig. 1, Supplementary Tables 1, 2). To explore the biological underpinnings, we computed the tissue enrichment for the closest genes to 545 individual variants from 395 loci with *p* values < 1e−5 from the nightmare GWAS and examined their expression in the GTEx data. This analysis showed a global enrichment of genetic loci active in the brain (*p* = 3.87e−5), potentially reflecting associations with sleep, mood and alcohol consumption as well as direct effects on nightmares themselves.

### Genetic analysis between nightmares and psychiatric traits

To explore how nightmares are related to previously reported epidemiological comorbidities and risk factors, we first examined the genetic correlation between PTSD [11] and the frequency of nightmares. We discovered a significant correlation (rg = 0.408, *p* = 0.0152) between frequent nightmares and PTSD, as expected from earlier literature. In addition, we observed a significant and robust genetic correlation of nightmares with a number of psychiatric traits (Table 1 for most significant traits, Supplementary Table 3 for all). We observed relatively different correlations between nightmares and psychiatric traits in the total nightmares at baseline population compared to the analysis of nightmares in which the individuals with heavy alcohol use were removed (Table 1).

**Table 1:** Genetic correlation from GWAS on the frequency of nightmares.

	Age, sex adjusted	Age, sex adjusted and heavy drinkers removed
Trait	rg (*p* value)	rg (*p* value)
Schizophrenia [42]	0.307	0.6124
	(8.585e−04)	(0.6971)
Depression [43]	0.5623	1.151
	(1.282e−07)	(0.3993)
Lifetime anxiety disorder UKB [44]	0.6436	1.446
	(8.234e−06)	(0.3839)
PTSD [11]	0.4083	−0.003016
	(0.01519)	(0.993)
Alcohol dependence [45]	0.2555	0.141
	(0.1932)	(0.7176)
Smoking Cessation [46]	0.247	0.5097
	(0.02968)	(0.4076)
Neuroticism [47]	0.6673	1.375
	(4.516e−07)	(0.4244)
Insomnia symptoms [35]	0.3936	0.6596
	(4.698e−05)	(0.3312)

The rg value computed by LDSC is not a bounded estimator and can range outside of −1 or 1.

To understand the relationship between nightmares and alcohol usage we used summary statistics for nightmares and nightmares without individuals who drink a lot of alcohol (these individuals were discounted from the GWAS). We used polygenic risk scores with nightmares to examine this connection further. We used polygenic risk scores to observe that scores of both populations were significantly associated with depressive symptoms (*p* = 5.36E−06), happiness (*p* = 7.39E−05), insomnia (*p* < 2.2E−16), and anxiety (seeing doctors for anxiety symptoms, *p* < 2.2E−16) in independent data from the UK Biobank (Table 2). We also tested against people seeking treatment for anxiety, which did not show a significant association (*p* > 0.05, Table 2). However, the score for nightmares adjusted for baseline covariates had slightly more explanatory power and more significant associations than the score for nightmares in which the individuals did not have an indication of heavy alcohol use (Table 2). Furthermore, the likelihood ratio test demonstrated a significant difference between the baseline model and the model in which individuals did not consume significant amounts of alcohol for all polygenic risk score tests (disorder ~ SCORE + age + sex) for all disorders in Table 2 (*p* < 2e−16).

**Table 2:** Polygenic risk score analysis of the baseline corrected nightmare cohort and nightmare cohort with individuals consuming significant amounts of alcohol removed.

	Beta	se	*p*	Adjusted R^2^
*Baseline corrected nightmares*				
Happiness (4526) ~ SCORE + age + sex + PC1–10	8.74E−03	2.40E−03	8.71E−05	7.90E−03
Happiness ~ age + sex + PC1–10	−8.30E−03	2.80E−04	<2.2E−16	7.77E−03
Depressed mood (2050) ~ SCORE + age + sex + PC1–10	5.90E−03	1.33E−03	9.14E−06	8.70E−03
Depressed mood ~ age + sex + PC1–10	−8.50E−03	1.67E−04	<2.2E−16	8.65E−03
Insomnia (1200) ~ SCORE + age + sex + PC1–10	1.33E−02	1.23E−03	<2.2E−16	2.95E−02
Insomnia ~ age + sex + PC1–10	8.99E−03	1.55E−04	<2.2E−16	2.92E−02
Anxiety-Seen doctor (2090) ~ SCORE + age + sex + PC1–10	8.12E−03	8.47E−04	<2.2E−16	2.36E−02
Anxiety-Seen doctor ~ age + sex + PC1–10	−2.01E−03	1.06E−04	<2.2E−16	2.34E−02
*Baseline corrected nightmares; individuals with significant alcohol consumption removed*				
Happiness (4526) ~ SCORE + age + sex + PC1–10	7.12E−03	2.23E−03	1.42E−03	7.85E−03
Happiness ~ age + sex + PC1–10	−8.30E−03	2.80E−04	<2.2E−16	7.77E−03
Depressed mood (2050) ~ SCORE + age + sex + PC1–10	4.66E−03	1.33E−03	4.64E−04	8.68E−03
Depressed mood ~ age + sex + PC1–10	−8.50E−03	1.67E−04	<2.2E−16	8.65E−03
Insomnia (1200) ~ SCORE + age + sex + PC1–10	1.07E−02	1.23E−03	<2.2E−16	2.94E−02
Insomnia ~ age + sex + PC1–10	8.99E−03	1.55E−04	<2.2E−16	2.92E−02
Anxiety-Seen doctor (2090) ~ SCORE + age + sex + PC1–10	6.06E−03	8.48E−04	8.87E−13	2.35E−02
Anxiety-Seen doctor ~ age + sex + PC1–10	−2.01E−03	1.06E−04	<2.2E−16	2.34E−02

SCORE signifies the Z-normalized polygenic risk score for the nightmare analysis. SE signifies standard error.

### Mendelian randomization between nightmares and psychiatric traits

To explore causality between nightmares and psychiatric traits, we performed a Mendelian randomization with a focus on phenotypes showing significant genetic correlation with the frequency of nightmares. Overall, insomnia showed significant causality as a risk factor for nightmares (nightmares baseline adjusted: beta = 0.027, *p* = 0.0002 and nightmares with individuals consuming more alcohol removed: beta = 0.02, *p* = 0.002), and no evidence of pleiotropy (MR intercept *p* > 0.05), whereas the other tested traits did not (Supplementary Table 4).

## Discussion

In this study, we explored the genetic underpinnings of nightmares, identifying genetic correlations between nightmares and psychiatric traits. In addition, the causal analysis suggests that insomnia increases the risk for frequent nightmares. Together with genetic correlations, these findings support the epidemiological findings that nightmares are an integral part of psychiatric traits, notably anxiety and depressive disorders as well as PTSD, and they increase after symptoms of insomnia.

An increased frequency of nightmares has been reported among patients with psychiatric disorders [2, 7, 10]. In the present study, we found significant genetic correlations between nightmares and mood disorders (major depression), anxiety disorders, and underlying personality characteristics (neuroticism). Furthermore, we identified a genetic correlation between nightmares and insomnia. This observation is consistent with epidemiological findings showing that nightmares correlate with sleep and psychiatric traits [2, 6, 8, 34]. However, our findings suggest not only an association but also a causal relationship between insomnia and the incidence of nightmares. Notably, the current genetic correlations are computed in nonoverlapping samples (Finnish population vs. UK Biobank) and do not have shared individuals. The genetic correlation or polygenic risk scores are therefore not induced by overlapping phenotypic representation in the same individuals who would be part of the discovery sample. As alcohol has been shown to have a potentially significant effect on nightmare incidence [6], and we had access to alcohol consumption data in our cohorts, we decided to test removing individuals that consume relatively high amounts of alcohol per week for a sensitivity analysis. Overall, the genetic correlation demonstrated a significant change in *p*-values between only baseline correction and removing individuals with heavy alcohol consumption (Table 1).

Symptoms of insomnia are very common and are reported by up to 30% of the population [35]. Insomnia has been linked to numerous psychological and physical disorders [36]. Furthermore, previous studies suggest a significant association between nightmares, sleep disorders, and psychiatric traits [37–39]. Overall, these results demonstrate that insomnia is a potential risk factor for increased nightmare frequency. However, this causal link should be examined more closely in a clinical setting.

One possible explanation for the comorbidity of nightmares and insomnia may be explained by the overlap between sleep complaints and nightmares, which was also seen in the present study. Nightmares usually occur during rapid eye movement (REM) sleep, while the instability of REM sleep accompanied by a slower resolution of emotional distress has been found to be of key importance in the pathophysiology of primary insomnia [15]. This finding could be one potential explanation for genetic and phenotypic correlations with nightmares. Our finding of shared genetic risk between nightmares and symptoms of insomnia would support this hypothesis.

A subset of our findings may reflect that some individuals have a higher capacity to recall dreams. For example, a higher nightmare recall frequency could be explained by a higher number of awakenings during the night, as putatively induced by fragmented sleep due to insomnia. Although the definition of a nightmare includes the awakening criterion, i.e., it is assumed that the dysphoric dream wakes up the dreamer, most individuals interpret nightmares simply as highly negative dreams and disregard the criterion for waking up. Additionally, in questionnaires, nightmares and the awakening criterion were not clearly defined for the participant. A higher dream and nightmare recall frequency could be explained by a higher number of awakenings during the night, because this frequency gives a greater opportunity to remember a dream upon awakening, including nightmares. Some of our observations support this reasoning. Accordingly, we observed increased nightmares in individuals reporting night-time awakenings. Notably, there was no genetic correlation with the number of sleep episodes.

In this study we found significant pleiotropy between nightmares and schizophrenia, and between nightmares and depression. These findings support pleiotropic effects between nightmares and psychiatric traits. However, we did not see pleiotropy with all tested traits. For example, there was no pleiotropy between insomnia and nightmares. We therefore recognize that the symptomatology of sleep and psychiatric traits and the exact clinical manifestation of nightmares within these traits should be carefully evaluated in future clinical, epidemiological and genetic studies.

The expression enrichment in the brain supports previous findings regarding sleep-associated traits [40]. Furthermore, larger studies on nightmares will potentially clarify the individual cell types in the brain that contribute to nightmares. As the enrichment method is an in silico analysis these observations will benefit from future experimental studies.

To summarize, these findings show two possible mechanisms for the association of the genetic risk for nightmare with insomnia, either a direct mechanism that is tied to sleep problems and the negativity of a dream or a modulatory effect that is associated with the frequency with which the dream is recalled. Furthermore, heavy alcohol consumption may modify the genetic correlations between nightmares and psychiatric traits. Given the significant correlations with psychiatric traits, it would be essential to grow awareness of how nightmares affect health and disease and systematically collect information about nightmares, especially in clinical samples, with a longitudinal follow-up, and then implement these results in larger cohorts. For example, by more routinely implementing questions for dream recall, nightmare frequency and distress, and nighttime habits. Our findings indicate the possibility that patients with psychiatric disorders may benefit if their nightmares are managed as part of the treatment strategy by using evidence-based interventions such as imagery rehearsal therapy [41].

### Supplementary information

Supplementary Table 1

Supplementary Table 2

Supplemetary Table 3

Supplementary Table 4

Supplementary Figure 1

## Data Availability

The summary statistics for these associations will be available at the Sleep Disorder Knowledge Port, http://sleepdisordergenetics.org/.
