# Supplementary material for: Nightmares share genetic risk factors with sleep and psychiatric traits
Source: Transl Psychiatry. 2024 Feb 27;14:123. doi: 10.1038/s41398-023-02637-6 (PMC10899618; doi:10.1038/s41398-023-02637-6)
Supplement: Supplementary file 1 — Supplementary Table 1 [file 41398_2023_2637_MOESM1_ESM.pdf]

Supplementary Table 1. Top 100 SNPs from the meta-analyses of the baseline-adjusted nightmares cohort.

SNP	ref	alt	Z-score	P-value
rs6723030	a	g	-5.063	4.12E-07
rs4523194	t	c	-5.008	5.50E-07
rs182628836	t	c	-4.965	6.88E-07
rs1832340	c	g	4.886	1.03E-06
rs61832415	t	g	-4.84	1.30E-06
rs11587851	t	c	4.823	1.41E-06
rs75875194	a	g	-4.823	1.42E-06
rs1362844	t	c	4.82	1.44E-06
rs12408892	t	c	4.799	1.59E-06
rs55924146	a	t	4.792	1.65E-06
rs10925347	a	t	-4.789	1.68E-06
rs10080901	t	c	4.777	1.78E-06
rs138188226	ca	c	4.775	1.80E-06
rs59610822	t	g	4.774	1.80E-06
rs11584450	a	g	4.771	1.84E-06
rs150556501	t	c	-4.768	1.86E-06
rs10802591	a	g	-4.734	2.20E-06
rs2326124	c	g	4.711	2.46E-06
rs72784254	t	c	4.707	2.51E-06
rs9302830	c	g	4.695	2.66E-06
rs10764616	c	g	-4.684	2.82E-06
rs56279830	t	c	-4.674	2.96E-06
rs7541818	a	g	4.66	3.16E-06
rs2241257	a	g	-4.652	3.28E-06
rs6665340	c	g	-4.65	3.33E-06
rs6661229	t	c	4.645	3.40E-06
rs17528774	t	c	4.644	3.41E-06
rs11076484	a	t	4.643	3.43E-06
rs72764084	a	t	-4.641	3.47E-06
rs72764083	t	c	-4.639	3.50E-06
rs2241255	c	g	-4.639	3.51E-06
rs187000018	t	c	-4.635	3.58E-06
rs12446591	a	g	4.63	3.65E-06
rs58933371	t	c	4.623	3.79E-06
rs12078693	c	g	-4.622	3.80E-06
rs537379350	c	g	-4.621	3.81E-06
rs1340467	t	g	-4.619	3.85E-06
rs1452118	a	c	4.612	3.99E-06
rs6683160	a	c	-4.611	4.01E-06
rs180948335	t	g	4.606	4.11E-06
rs35069948	a	g	4.603	4.17E-06
rs11801278	a	g	-4.601	4.20E-06
rs1978289	c	g	-4.6	4.22E-06
rs7541924	a	g	4.591	4.41E-06

rs12407646	t	c	4.585	4.53E-06
rs76449575	t	g	-4.546	5.47E-06
rs574921648	cattaa	c	4.544	5.53E-06
rs180878182	a	g	-4.543	5.54E-06
rs994850	a	t	4.542	5.57E-06
rs6703530	t	c	4.54	5.62E-06
rs111464894	t	c	4.533	5.81E-06
rs146026765	t	g	4.533	5.82E-06
rs7633256	t	g	-4.53	5.90E-06
rs56011612	a	g	-4.521	6.17E-06
rs574456506	t	g	4.517	6.26E-06
rs186980712	t	c	-4.516	6.31E-06
rs79206612	a	g	-4.509	6.52E-06
rs7576202	a	g	-4.495	6.95E-06
rs12129838	c	g	4.492	7.06E-06
rs8182494	t	g	-4.483	7.35E-06
rs4722277	t	c	-4.481	7.44E-06
rs312057	c	g	-4.473	7.72E-06
rs12446262	t	c	-4.462	8.12E-06
rs12426427	a	t	-4.458	8.27E-06
rs1920979	a	g	-4.449	8.65E-06
rs141331984	a	t	4.441	8.97E-06
rs1835623	t	g	4.441	8.97E-06
rs4468795	a	g	-4.44	8.99E-06
rs9373661	t	g	-4.439	9.04E-06
rs149706310	t	c	4.431	9.39E-06
rs139574033	a	c	4.431	9.40E-06
rs4946635	a	g	-4.429	9.47E-06
rs11860550	t	c	4.428	9.52E-06
rs58174773	a	g	-4.427	9.57E-06
rs10193201	c	g	-4.425	9.63E-06
rs10789446	t	c	-4.424	9.69E-06
rs76590349	t	c	4.423	9.72E-06
rs57703658	c	g	4.422	9.77E-06
rs12137834	t	c	4.421	9.83E-06
rs36157198	t	c	4.42	9.85E-06
rs7582092	a	g	-4.419	9.92E-06
rs1122435	a	g	-4.413	1.02E-05
rs7201200	t	g	-4.409	1.04E-05
rs35968313	a	g	4.407	1.05E-05
rs7753783	a	c	4.404	1.07E-05
rs9620627	a	g	-4.401	1.08E-05
rs6435362	t	c	4.395	1.11E-05
rs1453168	a	g	4.395	1.11E-05
rs10932174	a	g	4.394	1.11E-05
rs4749136	a	g	4.394	1.11E-05
rs146234551	cctttt	c	4.391	1.13E-05
rs79926321	t	c	-4.388	1.14E-05

rs13384272	t	c	-4.387	1.15E-05
rs61439131	a	t	4.378	1.20E-05
rs78844883	t	g	-4.377	1.21E-05
rs190348340	a	c	4.373	1.23E-05
rs185623996	a	g	-4.369	1.25E-05
rs73163292	t	c	-4.366	1.26E-05
rs7196550	a	g	4.359	1.31E-05
rs545507025	a	g	4.349	1.37E-05
