# Supplementary material for: Nightmares share genetic risk factors with sleep and psychiatric traits
Source: Transl Psychiatry. 2024 Feb 27;14:123. doi: 10.1038/s41398-023-02637-6 (PMC10899618; doi:10.1038/s41398-023-02637-6)
Supplement: Supplementary file 2 — Supplementary Table 2 [file 41398_2023_2637_MOESM2_ESM.pdf]

Supplementary Table 2. Top 100 SNPs from the meta-analyses of the baseline-adjusted nightmares cohort and the cohort from which individuals consuming significant amounts of alcohol have been removed.

SNP	ref	alt	Zscore	P-value
rs138188226	ca	c	5.263	1.42E-07
rs6723030	a	g	-5.225	1.74E-07
rs10080901	t	c	5.08	3.77E-07
rs537379350	c	g	-5.034	4.80E-07
rs11910752	a	g	-5.009	5.48E-07
rs556984107	t	c	4.947	7.55E-07
rs2789678	t	c	4.932	8.14E-07
rs75875194	a	g	-4.858	1.18E-06
rs4523194	t	c	-4.847	1.25E-06
rs7467075	a	t	-4.816	1.47E-06
rs61908391	t	c	-4.808	1.52E-06
rs75521460	a	g	-4.781	1.75E-06
rs12426427	a	t	-4.757	1.96E-06
rs146344766	g	gc	-4.72	2.36E-06
rs731243	t	c	-4.672	2.98E-06
rs6141764	t	c	-4.662	3.13E-06
rs73334618	a	c	4.649	3.34E-06
rs9983870	t	c	-4.645	3.40E-06
rs60698335	t	c	-4.64	3.48E-06
rs731242	a	c	-4.633	3.60E-06
rs78320184	t	c	-4.63	3.66E-06
rs75466795	t	c	4.626	3.72E-06
rs4342283	a	g	-4.619	3.85E-06
rs73334614	a	g	4.616	3.90E-06
rs1833748	a	g	4.6	4.23E-06
rs2910244	a	g	4.583	4.58E-06
rs72707091	t	c	-4.582	4.60E-06
rs9979991	t	c	4.563	5.05E-06
rs187877854	a	g	-4.547	5.45E-06
rs951918	a	g	-4.544	5.53E-06
rs2202111	t	c	-4.543	5.55E-06
rs330453	a	g	4.522	6.14E-06
rs73334610	t	c	-4.516	6.31E-06
rs716467	a	g	4.515	6.34E-06
rs77008438	a	c	4.514	6.37E-06
rs539817541	a	g	4.513	6.39E-06
rs2546855	a	g	-4.508	6.55E-06
rs2239387	t	c	-4.504	6.66E-06
rs148411066	ct	c	-4.502	6.74E-06
rs113512623	a	g	-4.5	6.79E-06
rs62062201	t	c	4.499	6.81E-06
rs7530247	t	c	-4.497	6.91E-06
rs6797171	a	g	-4.49	7.14E-06

rs28759836	a	t	-4.487	7.22E-06
rs1363662	a	g	-4.476	7.60E-06
rs28857337	t	g	-4.467	7.95E-06
rs4832810	t	c	4.462	8.13E-06
rs2859487	t	c	-4.456	8.34E-06
rs10660436	a	aggtg	4.448	8.65E-06
rs716466	a	g	-4.448	8.68E-06
rs4369201	t	g	-4.448	8.68E-06
rs146234551	cctttt	c	4.443	8.85E-06
rs1340467	t	g	-4.443	8.88E-06
rs2303847	t	g	4.436	9.17E-06
rs2652264	t	c	-4.432	9.35E-06
rs1217753	t	c	-4.411	1.03E-05
rs115736519	t	c	4.407	1.05E-05
rs148215404	t	g	-4.404	1.06E-05
rs9874801	a	g	-4.391	1.13E-05
rs10737389	a	g	-4.39	1.14E-05
rs62426154	a	c	-4.386	1.15E-05
rs117210054	a	g	4.38	1.19E-05
rs73163365	t	c	-4.355	1.33E-05
rs78477743	t	c	4.348	1.37E-05
rs7728135	a	g	-4.345	1.39E-05
rs140946170	a	c	-4.344	1.40E-05
rs188219558	a	g	-4.343	1.41E-05
rs185014484	t	g	-4.332	1.48E-05
rs571415717	a	g	-4.332	1.48E-05
rs193031790	t	g	-4.326	1.52E-05
rs330451	t	c	-4.325	1.52E-05
rs34537844	t	g	-4.319	1.57E-05
rs75948041	a	t	-4.316	1.59E-05
rs117750034	t	c	-4.316	1.59E-05
rs13094855	a	t	4.313	1.61E-05
rs200243126	cctggg	c	-4.308	1.65E-05
rs117845249	t	c	-4.304	1.68E-05
rs148026361	a	c	-4.303	1.68E-05
rs7460996	a	g	4.303	1.69E-05
rs140163472	t	g	4.298	1.73E-05
rs167928	t	g	-4.297	1.73E-05
rs62426152	a	g	4.296	1.74E-05
rs13077372	t	c	-4.293	1.76E-05
rs3744410	t	g	-4.292	1.77E-05
rs12236389	a	g	4.291	1.78E-05
rs1605030	a	t	4.288	1.80E-05
rs13078063	a	g	4.288	1.81E-05
rs184409726	a	g	-4.286	1.82E-05
rs1869090	t	c	4.285	1.83E-05
rs75732871	t	c	4.285	1.83E-05
rs6545696	t	c	-4.282	1.86E-05

rs35919500	a	g	-4.281	1.86E-05
rs4982558	a	g	-4.277	1.89E-05
rs4312919	a	g	-4.275	1.91E-05
rs4491881	t	g	4.275	1.91E-05
rs416571	a	g	4.274	1.92E-05
rs17684675	c	g	-4.272	1.93E-05
rs1847836	a	g	-4.272	1.94E-05
rs36010116	a	g	-4.271	1.94E-05
rs56242841	t	c	4.271	1.95E-05
