# Supplementary material for: Nightmares share genetic risk factors with sleep and psychiatric traits
Source: Transl Psychiatry. 2024 Feb 27;14:123. doi: 10.1038/s41398-023-02637-6 (PMC10899618; doi:10.1038/s41398-023-02637-6)
Supplement: Supplementary file 4 — Supplementary Table 4 [file 41398_2023_2637_MOESM4_ESM.pdf]

Supplementary Table 4. Mendelian randomization results, demonstrating significance for insomnia as a risk factor for nightmares.

Nightmares BL corrected

**Exposure**	**nSNPs**	**IVW beta**	**IVW se**	**IVW P-value**	**MR Intercept**
Insomnia	233	0.027058	0.007169	0.000161	0.47
Depression	50	-0.02416	0.022505	0.282936	0.45
Alcohol use disorder	9	0.158192	0.213538	0.458804	0.49
Alcohol consumption	83	-0.01275	0.049944	0.798465	0.39
Neuroticism	8	-0.15245	0.100278	0.128438	0.26
Schizophrenia	104	-0.01351	0.007516	0.072352	0.015

Nightmares (individuals with significant alcohol consumption removed)

**Exposure**	**nSNPs**	**IVW beta**	**IVW se**	**IVW P-value**	**MR Intercept**
Insomnia	233	0.022712	0.007509	0.002491	0.53
Depression	52	0.001539	0.024436	0.949791	0.047
Alcohol use disorder	9	0.069087	0.259472	0.79004	0.52
Alcohol consumption	83	-0.06248	0.053903	0.246426	0.92
Neuroticism	8	-0.16354	0.105315	0.120464	0.36
Schizophrenia	107	-0.00457	0.007785	0.556943	0.006
