# Supplementary figures and images for: Nightmares share genetic risk factors with sleep and psychiatric traits
Source: Transl Psychiatry. 2024 Feb 27;14:123. doi: 10.1038/s41398-023-02637-6 (PMC10899618; doi:10.1038/s41398-023-02637-6)

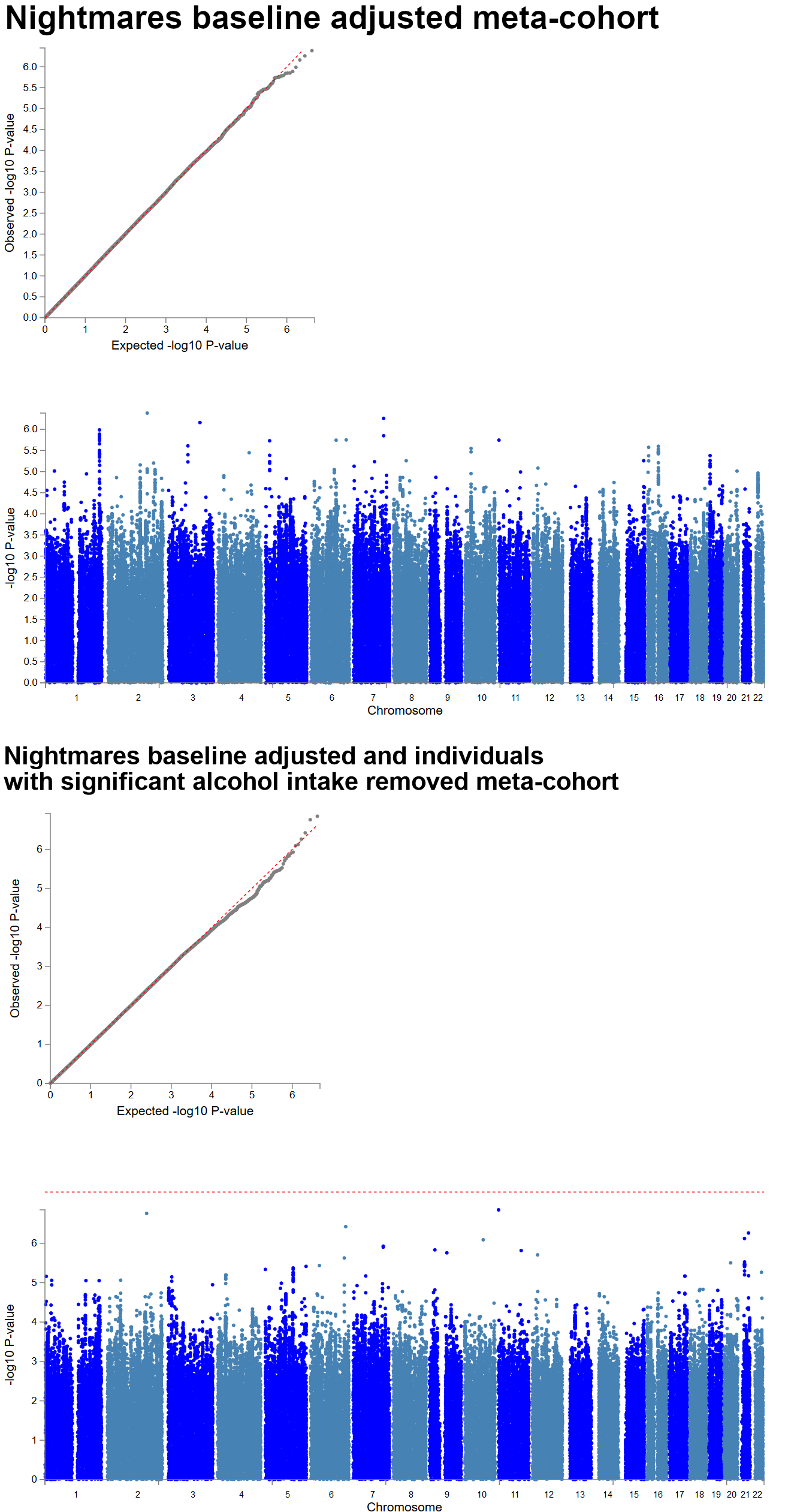

Supplement: Supplementary file 5 — Supplementary Figure 1 [file 41398_2023_2637_MOESM5_ESM.tiff]
